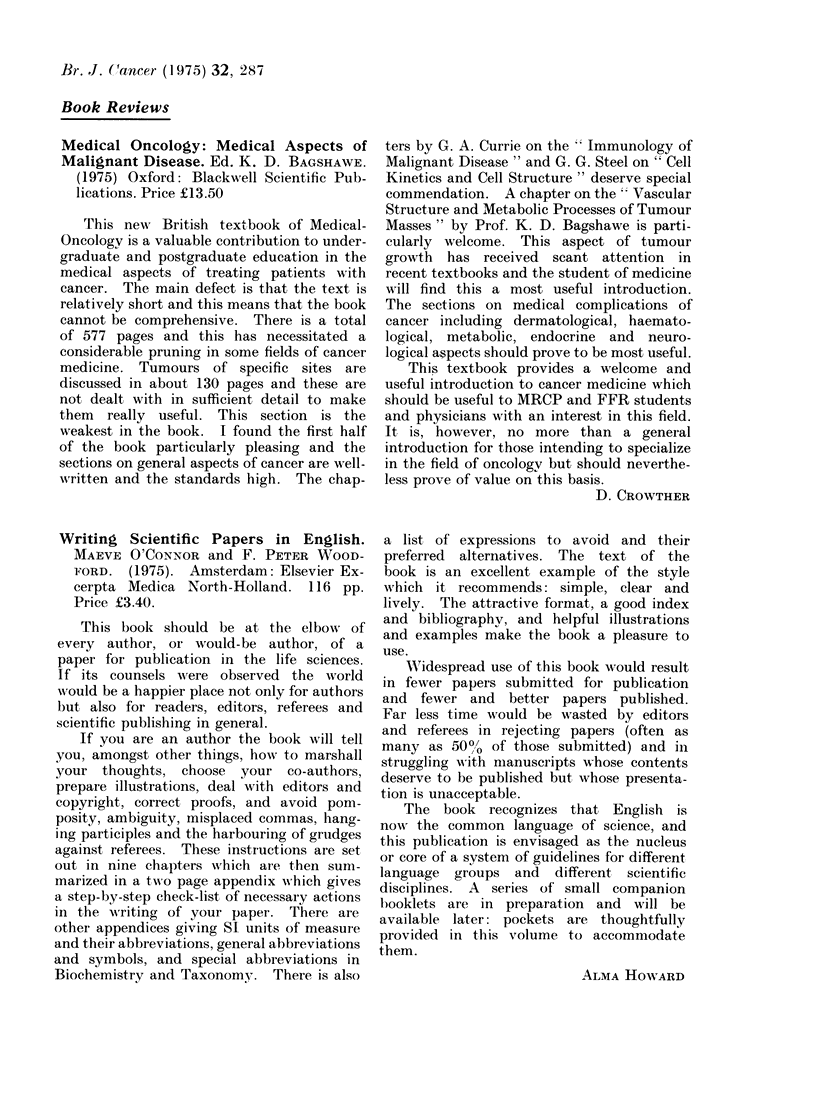# Writing Scientific Papers in English

**Published:** 1975-08

**Authors:** Alma Howard


**Writing Scientific Papers in English.** MAEVE O'CONNOR and F. PETER WOODFORD. (1975). Amsterdam: Elsevier Excerpta Medica North-Holland. 116 pp. Price £3.40.

This book should be at the elbow of every author, or would-be author, of a paper for publication in the life sciences. If its counsels were observed the world would be a happier place not only for authors but also for readers, editors, referees and scientific publishing in general.

If you are an author the book will tell you, amongst other things, how to marshall your thoughts, choose your co-authors, prepare illustrations, deal with editors and copyright, correct proofs, and avoid pomposity, ambiguity, misplaced commas, hanging participles and the harbouring of grudges against referees. These instructions are set out in nine chapters which are then summarized in a two page appendix which gives a step-by-step check-list of necessary actions in the writing of your paper. There are other appendices giving SI units of measure and their abbreviations, general abbreviations and symbols, and special abbreviations in Biochemistry and Taxonomy. There is also a list of expressions to avoid and their preferred alternatives. The text of the book is an excellent example of the style which it recommends: simple, clear and lively. The attractive format, a good index and bibliography, and helpful illustrations and examples make the book a pleasure to use.

Widespread use of this book would result in fewer papers submitted for publication and fewer and better papers published. Far less time would be wasted by editors and referees in rejecting papers (often as many as 50% of those submitted) and in struggling with manuscripts whose contents deserve to be published but whose presentation is unacceptable.

The book recognizes that English is now the common language of science, and this publication is envisaged as the nucleus or core of a system of guidelines for different language groups and different scientific disciplines. A series of small companion booklets are in preparation and will be available later: pockets are thoughtfully provided in this volume to accommodate them.

ALMA HOWARD